# Experimental study on the effects of water content on the compression characteristics and particle breakage of calcareous sand

**DOI:** 10.1038/s41598-024-57505-0

**Published:** 2024-03-21

**Authors:** Xiaoxuan Liu, Xingxiao Wang, Xiaobing Wei, Mingxing Luo, Xinlian Chen, Li Zhong

**Affiliations:** 1 School of Civil Engineering and Architecture, Wuhan University of Technology, Wuhan, 430070 Hubei China; 2 School of Civil Engineering, Hubei Engineering University, Xiaogan, 432000 Hubei China; 3 Hubei Small Town Development Research Center, Hubei Engineering University, Xiaogan, 432000 Hubei China; 4 Beijing Xinyi Resources Technology Co., Ltd, Zhengzhou, 450000 Henan China; 5 Zhejiang Zhongjiao Tongli Engineering Design Co., Ltd, Hangzhou, 310000 Zhejiang China

**Keywords:** Calcareous sand, Particle breakage, Water content, Compression characteristics, Ocean sciences, Civil engineering, Mechanical engineering

## Abstract

The particle breakage effect and compression characteristics of calcareous sand are related to the water content in the sand material. However, the effects of water content on the particle breakage and compression characteristics of calcareous sand have rarely been investigated. In this work, 50 sets of confined compression tests were conducted on calcareous sand specimens, and the compression characteristics and particle breakage effects of two single-particle-size groups (particle size ranges of 1–0.5 mm and 0.5–0.25 mm) of calcareous sand were investigated under five different water contents. The test results showed that with the increase in the water content, the final compression deformation of calcareous sand was positively correlated with the water content. The final compression deformation decreased when the water content reached a certain value. The water content corresponding to the peak final compression deformation was related to the gradation of the calcareous sand; the specific values were 10% and 15% for particle size ranges of 1–0.5 mm and 0.5–0.25 mm, respectively. With the increase in the water content, the slope of the loading curve of calcareous sand appeared to increase and then decrease, reaching maximum when the water content was 10%. Moreover, the slope of the loading curve was close to twice that of the loading curve of dry sand, whereas the slope of the unloading curve changed little. Under the same water content, the initial gradation had no effect on the compression and unloading characteristics of the specimens beyond a vertical pressure of 1 MPa. The effects of the variation in the water content on the particle breakage of calcareous sand were mainly reflected in the softening effect of water on the specimen particles, which reduced the Mohr strength of the particles.

## Introduction

Calcareous rock and soil is a unique type of rock and soil material formed after the death of marine calcareous organisms, such as reef-building coral communities (e.g., corals, seaweeds, shellfish), and under the long-term effect of geologic (e.g., proximal transport deposition, etc.) and physicochemical factors (e.g., breakage of debris, calcification, etc.). It is more commonly found close to the continental shelves of tropical or sub-tropical climates and along coastlines (generally between 30° N and 30° S). Particle breakage of calcareous sand under a constant stress is a distinctive characteristic of new geotechnical media^1,2^. Macroscopically, particle breakage directly leads to a change in the particle gradation (composition), which causes a decrease in the void ratio and an increase in the relative density of calcareous sand, and has non-negligible effects on its strength (internal friction angle), dilatancy, critical state, compression deformation properties, and permeability coefficient, resulting in a variation in the constitutive relationship of calcareous sand. Microscopically, the shear strength of calcareous sand is mainly related to the magnitude of the friction between the particles and the contact relationship between the particles. Particle breakage leads to a change in the particle shape, causing a variation in the particle friction coefficients as well as rearrangement of particle positions, which affects the contact relationships between particles and also the microstructure. Hence, particle breakage is a key factor in studying the mechanical properties of calcareous sand.

Experimental and quantitative analyses have shown that many factors affect the particle breakage of calcareous sand^3–5^. For example, Coop and Sorensen^2^ conducted ring shear tests on calcareous sand and found that particle breakage continues to occur under large strains, and the final particle size distribution was related to the initial gradation and confining pressure. Miao and Airey^6^ analyzed the effects of the variations in the void ratio, gradation property, and particle shape on the particle breakage of calcareous sand through large-strain ring shear tests and high-pressure consolidation tests. Xiao et al.^7^ conducted confined compression tests on calcareous sand at vertical pressures in the range 0.1–3.2 MPa and found that particle breakage as well as volumetric deformation of calcareous sand are directly proportional to the input work and that the relationship between particle breakage and the input work is independent of the initial density. Liu et al.^8^ conducted a series of confined compression tests and triaxial tests under different test conditions on two types of calcareous sand specimens collected from the South China Sea. The results showed that a significant amount of particle breakage occurs in calcareous sand under different loading modes, significantly affecting its mechanical properties. Moreover, an increase in the confining pressure led to an increase in the particle breakage. Through stress path tests on limestone rockfill material, Xu et al.^9,10^ found that the stress level and loading path are key factors affecting the stress–strain relationship of soil. A DEM simulation revealed that the confining pressure, deviatoric stress, and loading direction significantly influence the evolution of the breakage time and breakage mode of aggregates during shearing. Liu^11^, Wu^12^, Hassanlourad^13^, Brandes^14^, and Shahnazari^15^ proposed factors affecting the particle breakage of calcareous sand through experiments or directly introduced the influencing factors into the constitutive relationships of calcareous sand^16,17^.

However, most of these studies have been based on the effects of various factors on the particle breakage of calcareous sand in the saturated state. Theoretically, the water content is another key factor affecting the compression characteristics and particle breakage of calcareous sand. The compression properties of calcareous sand are similar to those of normally consolidated cohesive soils^18,19^, the internal pores of which reflect the water content capacity of sand specimens. The three-phase proportionality relationship directly affects the physical state and indirectly reflects the engineering properties of calcareous sand. Therefore, the water content and compression properties of calcareous sand have some theoretical relationship with particle breakage. Moreover, water plays a lubricating role in the relative movement between particles, and different water contents can cause different degrees of particle breakage in granular soil. Many researchers have confirmed that particle breakage increases with the increase in the water content. For example, Marsal^20^ and Chavez et al.^21^ experimentally demonstrated that granular soil with a high water content is more susceptible to breakage. Oldecop and Alonso^22^ explained that this phenomenon is due to the corrosive effect of water on the particles, which causes internal softening of the particles and makes a wet specimen to be more fragile than a dry specimen. Nieto-Gamboa^23^ showed that the effective capillary tensile stress in the internal cracks of particles increases because of the increase in the water content, exacerbating crack expansion and particle breakage. Xiao et al.^24^ conducted direct box shear tests on silica sand with different dry densities and water contents to investigate the effects of dry density and water content on the shear strength of silica sand. The stress required to achieve the same strain in the standard sand specimen was found to increase with the increase in the dry density of the standard sand and the normal stress of the test, whereas it decreased with the increase in the water content of the standard sand. Li et al.^25^ reached similar conclusions through tests. Liu et al.^26^ experimentally found a correlation between the water content and thermal conductivity of calcareous sand. However, the effects of water content on the compression characteristics and particle breakage of calcareous sands are yet to be explored.

Considering that the water content of calcareous sand is closely related to the particle breakage and compression characteristics and taking calcareous sand from the sea near a reef in the Nansha Islands of China as the test material, 50 sets of confined compression tests were conducted in this study under different gradations, water contents, and vertical pressures (tests in which the specimens were laterally restricted so that no lateral deformation could occur, and compression deformation was produced only in the vertical direction). The compaction characteristics and particle breakage law of calcareous sand with different water contents under different vertical loads were investigated. The novelty of this study was to analyze the relationship between the water content and particle breakage and compression characteristics. The findings can provide the necessary theoretical basis and reference for engineering practice in the construction of islands and reefs as well as in integrated ocean management.

## Materials and test methods

### Test instrument

**Figure 1:**
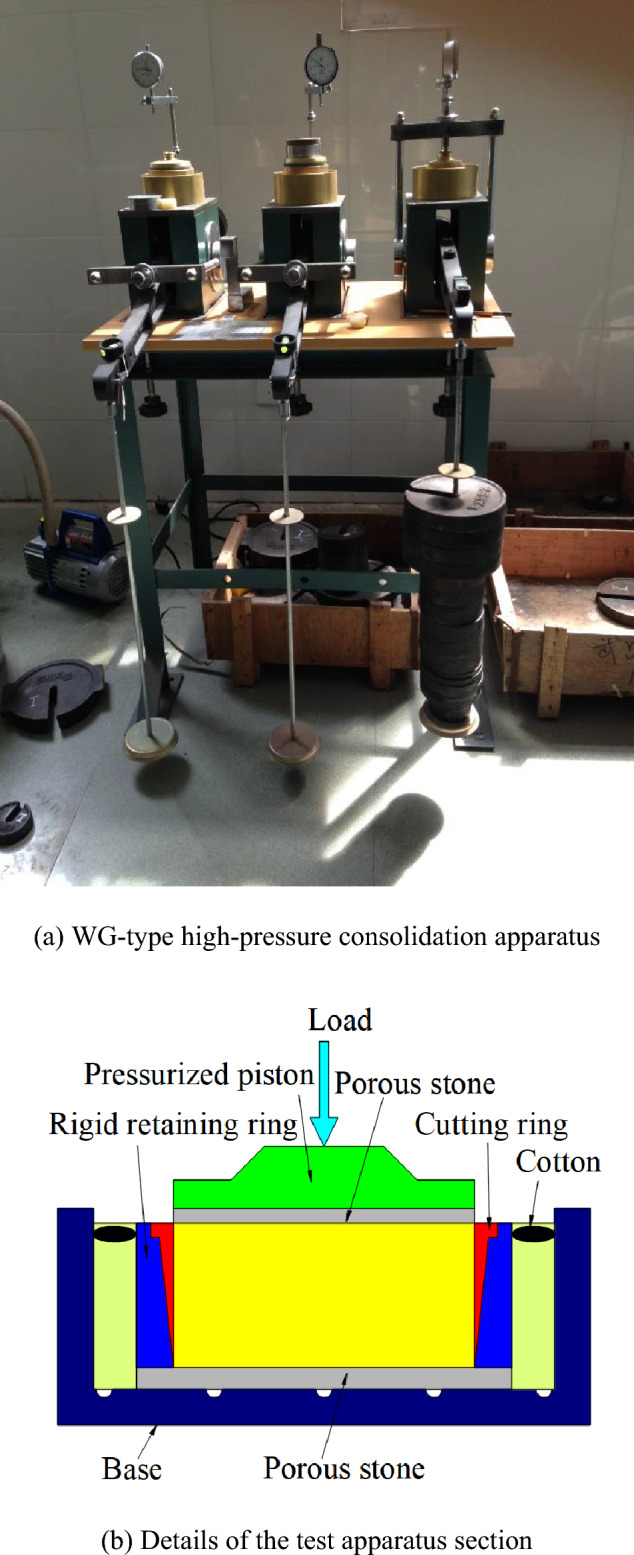
WG-type high-pressure consolidation apparatus.

The test apparatus was a WG-type high-pressure consolidation apparatus produced by Nanjing Instrument and Equipment Factory. The cutting ring of the device specimen was a steel mold with a diameter of 6.18 cm and a height of 2 cm. The vertical loading in the test was implemented using weights with a maximum vertical stress of 4000 kPa. The vertical deformation was measured using a dial indicator with a range of 10 mm, placed on a loading bar, as illustrated in Fig. 1a. Figure 1b shows the section of the test apparatus.

### Test material

**Figure 2:**
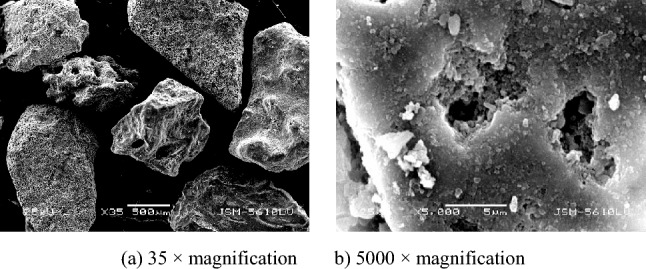
Scanning electron microscopy (SEM) images of Specimen B at different magnifications.

**Figure 3:**
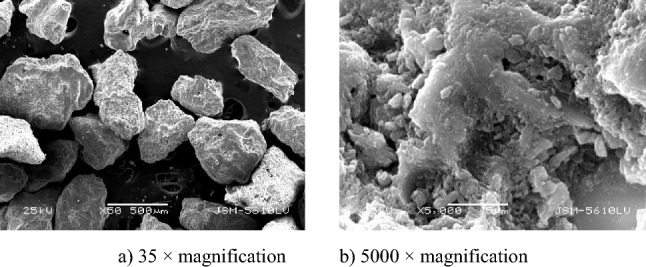
Scanning electron microscopy (SEM) images of Specimen S at different magnifications.

Calcareous sand from the sea near Yongshu Reef, Nansha Islands was used as the test material. The calcareous sand was qualitatively and quantitatively analyzed by X-ray fluorescence spectrometry. Table 1 presents the test results. The key constituent of the test material was calcium carbonate. As revealed by an X-ray fluorescence spectrometry analysis, calcium carbonate accounts for 96.39% of the total content. The specific gravity of the particles was 2.75. The sand specimens were washed with water and air-dried for use. Previous studies have revealed that the particle breakage of calcareous sand with a single particle size is greater than that of calcareous sand with a continuous gradation, and that the particle shape affects the particle breakage and compression characteristics of calcareous sand. To increase the particle breakage effects and eliminate the effects of particle shape, two particle sizes in the ranges of 1–0.5 mm (Specimen B) and 0.5–0.25 mm (Specimen S) were selected through a sieving test. Figures 2 and 3 show the scanning electron microscopy (SEM) images of the two calcareous sand specimens with two different particle sizes. The fine morphology of the sand specimens revealed that the sand specimen particles have an uneven surface, many fissure defects, evident angles, low roundness, high angularity, and rich internal pores.

**Table 1:** Contents of each component in calcareous sand (%).

Composition	SiO_2_	Al_2_O_3_	Fe_2_O_3_	CaO	MgO	Na_2_O	SO_3_	P_2_O_5_	SrO	Cl	Ignition loss
Content/%	0.13	0.056	0.03	53.12	1.70	0.45	0.40	0.064	0.66	0.028	43.27

### Test method

To investigate the particle breakage under different water contents and stresses, five specimens were preset with different water contents in the test: air-dried sand specimens (0.6%, 5%, 10%, and 15%) and saturated sand specimens (42%). The preset initial void ratio was 1.2. Except for the saturated specimens, which were prepared from air-dried sand specimens according to the preset void ratios using pumping saturation, the preset water content was taken for the rest of the sand specimens. The required sand specimens were weighed based on the preset void ratio and the volume of the cutting ring, and the specimens were placed into the cutting ring using the air pluviation method^27^.

The loading pressures for test termination were 1200, 1600, 2400, 3200, and 4000 kPa, and the unloaded rebound test was performed at 4000 kPa. The test was strain-controlled. The stabilization criterion for each loading level was that the variation in the dial gauge reading should not exceed 0.01 mm per hour, and the next level of loading was applied until the predetermined level of the termination pressure was reached. After the test, the specimens were carefully removed, dried and weighed, and finally sieved. Considering the low strength of calcareous sand particles and the possibility of particle breakage during sieving, which can affect the test results, the sieving time was set to 15 min. Table 2 presents the specific schemes of the tests.

**Table 2:** Test schemes.

Test	Specimen	Initial water content (%)	Consolidation pressure *σ*_h_/MPa	Number of tests
B1	Specimen B	0.6	1.2/1.6/2.4/3.2/4.0	5
B2		5	1.2/1.6/2.4/3.2/4.0	5
B3		10	1.2/1.6/2.4/3.2/4.0	5
B4		15	1.2/1.6/2.4/3.2/4.0	5
B5		42^1^	1.2/1.6/2.4/3.2/4.0	5
S1	Specimen S	0.6^2^	1.2/1.6/2.4/3.2/4.0	5
S2		5	1.2/1.6/2.4/3.2/4.0	5
S3		10	1.2/1.6/2.4/3.2/4.0	5
S4		15	1.2/1.6/2.4/3.2/4.0	5
S5		42	1.2/1.6/2.4/3.2/4.0	5

^1^The water content of the saturated specimen is approximately 42%

^2^The water content of the air-dried sand specimen is 0.6%

For each tested specimen, the particle size distribution curves after the test were obtained using the sieve analysis method. According to previous studies^28,29^, the relative breakage index *B*_r_ reported by Hardin^30^ was selected as the breakage index in this study. The specific steps are as follows: the areas enclosed by the particle size distribution curves before and after the test and the 0.075 mm particle size cut-off line were calculated. The particle breakage index was the ratio of the difference between the two areas and the area enclosed by the particle size distribution curve before the test and the 0.075 mm particle size cut-off line.

## Results and analysis

### Particle breakage under different water contents

**Figure 4:**
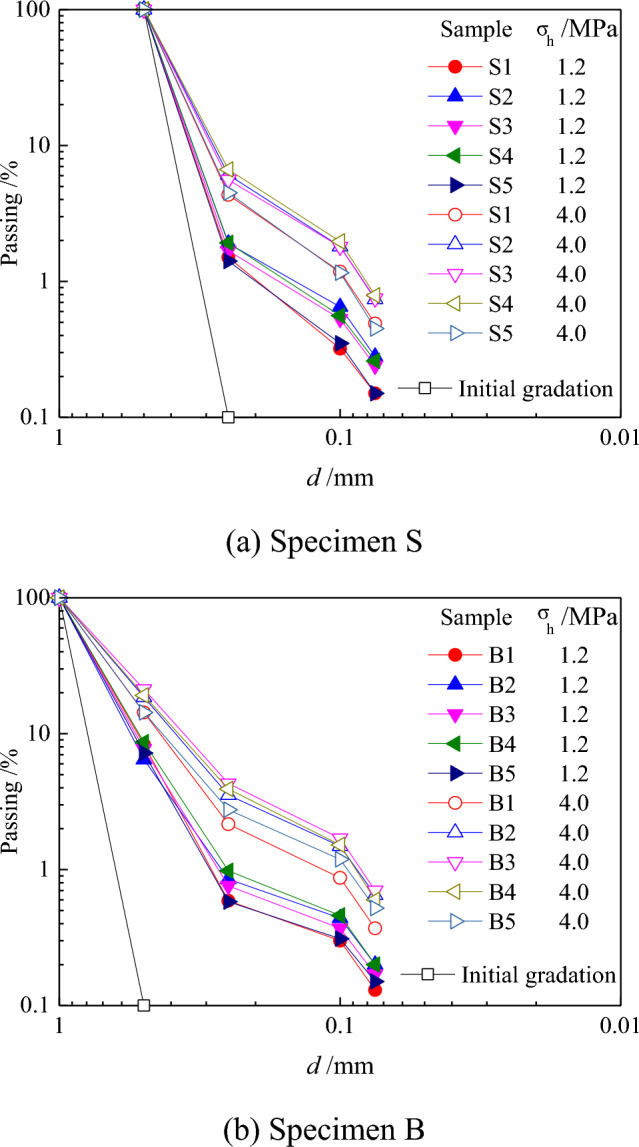
Gradation curves under different vertical pressures.

Figure 4 shows the particle size distribution curves before and after the test under different water contents at 4 and 1.2 MPa (for the convenience of drawing in the figure, the original particle size distribution curve is plotted as 0.1% when the content is zero). The calcareous sand specimen exhibited evident particle breakage under pressure. The greater the pressure, the more evident the breakage phenomenon. Each specimen was crushed under pressure, and although the fine particles increased in large quantities, the initial particle size of both specimens S and B was retained in large quantities. This is consistent with the breakage phenomenon of quartz sand particles reported by Zhang et al.^31^. This can be explained by the fact that during the consolidation process, the resistance of larger particles is enhanced due to the buffering of the surrounding small particles.

As shown in Fig. 4, the particles have different degrees of growth for different water contents under a vertical pressure. Taking Specimen B as an example, a detailed analysis of its particle breakage revealed that all the particle size groups except for those with a particle size range of 1–0.5 mm showed different degrees of growth compared with the original gradation. This indicated that a certain particle breakage occurred in the particle size range of 1–0.5 mm under loading. The variation in each curve was not significant: the growth of particles in the size range of 1–0.5 mm decreased, whereas the growth of particles in other size ranges increased, and there was no evident growth of particles with sizes below 0.5 mm. Clearly, although particle breakage occurred in the compression process of Specimen B, the growth of particle breakage was not evident, and the particle breakage was dominated by particles in the size range of 1–0.5 mm. Even under maximum loading, particles in the size range of 1–0.5 mm decreased considerably but did not completely disappear. The content of particles in the size range of 0.5–0.25 mm decreased compared with the upper-level loading test, indicating that some of the particles in the size range of 0.5–0.25 mm were broken into particles smaller than 0.25 mm during the compression process. A comparison of Specimens B and S without considering the effects of the differences in the gradation parameters and under the same termination vertical pressure showed that due to the closer contact between particles with a lower average particle size (Specimen S), the surface of the coral sand particles (Specimen B) relative to that of the coarse particles did not have any evident defects and had more internal pores. Moreover, coral sand with a relatively small average particle size (Specimen S) produced less particle breakage under external force. The coarse particle group (Specimen B) could be characterized by irregularly shaped particles, such as branches and coral fragments, and the particles were often sharp-edged. The particles were larger, the effective contact points of the particles were fewer, and these edges were prone to produce stress concentration that led to brittle particles, which resulted in a higher breakage index for coral sand with a larger average particle size (Specimen B). A small amount of water infiltrated the soil particles, resulting in a capillary suction between the particles due to surface tension. This effect hindered the normal movement between the particles and increased the inter-particle sliding resistance, resulting in a higher breakage of wet sand particles than dry sand particles in the compression process.

**Figure 5:**
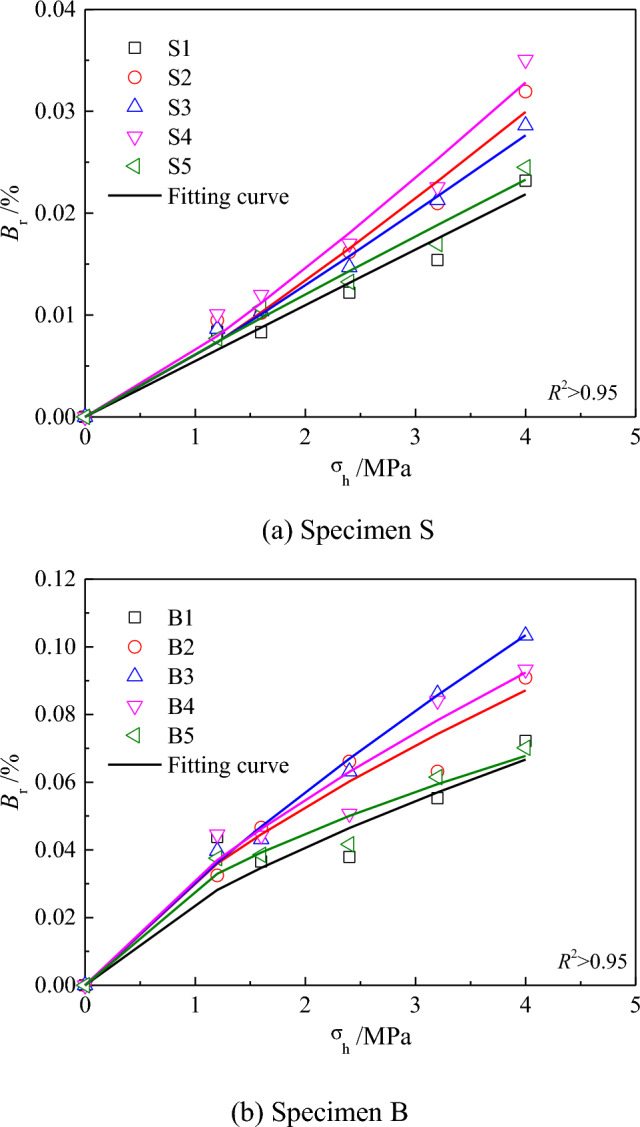
Vertical pressure versus the relative breakage index.

Figure 5 shows the curves of the particle breakage index versus the vertical pressure under different water contents. The particle breakage index increased with the increase in the confining pressure. The relationship between the particle breakage index and confining pressure presented the following functional relationship.

1 $$B_{r} = \frac{{\left( {\sigma /K_{b} } \right)^{{n_{a} }} }}{{1 + \left( {\sigma /K_{b} } \right)^{{n_{a} }} }}.$$

Here, *σ* denotes the vertical stress, and *B*_r_ denotes the relative breakage index. *K*_b_ and *n*_a_ denote the fitting parameters.

It is easy to understand the relationship between the aforementioned relative breakage index and vertical stress. When the vertical stress was equal to zero, the relative breakage index was zero. When the vertical pressure tended to infinity, the particles were all crushed to a size less than 0.074 mm, at which point the relative breakage index reached a limiting value of 100%. The values of *K*_b_ and *n*_a_ at each water content could be obtained by fitting, as listed in Table 3. The relative breakage index of calcareous sand under different water contents and vertical stresses could be calculated using Eq. (1) and the fitting curve (as shown in Fig. 5). As shown in Table 3, different water contents had different effects on the two fitting parameters, and the fitted parameter *K*_b_ was evidently more sensitive to the variation in the water content than *n*_a_.

**Table 3:** Comparison of fitting parameters.

Water content	Fitting parameters of Specimen S		Fitting parameters of Specimen B	
	*K*_b_	*n*_a_	*K*_b_	*n*_a_
Dry sand	172.52	1.01	134.86	0.75
5%	74.38	1.19	81.31	0.78
10%	96.11	1.12	40.79	0.93
15%	67.07	1.20	67.14	0.81
Saturated	188.40	0.97	256.56	0.63

Hardin^30^ proposed a similar relational equation in 1985 as follows:

2 $$B_{r} = \frac{{\left( {{{\sigma_{b} } \mathord{\left/ {\vphantom {{\sigma_{b} } {\sigma_{r} }}} \right. \kern-0pt} {\sigma_{r} }}} \right)^{{n_{b} }} }}{{1 + \left( {{{\sigma_{b} } \mathord{\left/ {\vphantom {{\sigma_{b} } {\sigma_{r} }}} \right. \kern-0pt} {\sigma_{r} }}} \right)^{{n_{b} }} }},$$

3 $$\sigma_{r} = \frac{{h^{2} 800p_{a} }}{{\left( {1 + e_{i} } \right)n_{s} }}.$$

Here, *σ*_b_ denotes the effective stress of breakage, and *σ*_r_ denotes the baseline breakage stress, which reflects the ability of the particles to resist breakage. *h* denotes the Mohr strength of the particles, and *n*_b_ denotes the particle breakage index. *p*_a_ denotes the standard atmospheric pressure, *e*_i_ denotes the initial void ratio, and *n*_s_ denotes the particle shape coefficient.

Comparing Eqs. (1) and (2), it can be found that the fitting parameter *K*_b_ can reflect the ability of the particles to resist breakage, which is correlated with the Mohr strength *h* of the particles. This indicated that the effects of the variation in the water content on the particle breakage of calcareous sand were mainly reflected in the softening effect of water on the specimen particles. This reduced the Mohr strength of the particles, which was macroscopically reflected in the relative breakage index of the particles. As listed in Table 3, this softening effect is not completely positively correlated with the water content.

The fitting parameter *n*_a_ under the same particle size did not vary significantly, whereas the fitting parameter *n*_a_ under different particle sizes had a large variability, indicating that the effects of the variation in the gradation on the relative breakage index can be expressed by *n*_a_.

### Compression characteristics of sand specimens with different water contents

**Figure 6:**
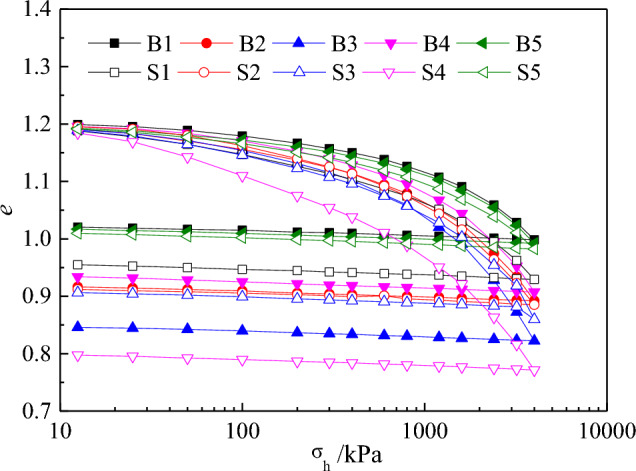
Loading–unloading compression curves of calcareous sand.

Figure 6 shows the loading–unloading compression curves of Specimens B and S at various water contents. The compression curves exhibited an upward convex shape under all pressure levels, and there was a significant consolidation yield pressure. However, the significant variability in the compression curves under different water contents indicated that the compression characteristics of the calcareous sand were sensitive to the water content. With the increase in the water content, the final compression deformation of the calcareous sand gradually increased. However, when the water content reached a certain value, the final compression deformation decreased. For Specimen B, this occurred at a water content of 10%, and for Specimen S, this occurred 15%, which may have been due to the unique internal pores in the calcareous sand (e.g. Fig. 3).

During the test, although the water content gradually increased, the surface of the calcareous sand did not contain much water (except in the saturated specimens), and the water was essentially stored within the internal pores of the calcareous sand. With the increase in the pressure, the particles moved, crushed, rearranged, and accordingly accumulated, and the water in the internal pores was squeezed and released. The capillary water pressure and surface tension of the water intensified the agglomeration of particles between the pores, and the final compression deformation increased^23^. However, with the increase in the water content of calcareous sand, the compression of the sand specimen gradually changed from the two-phase medium of the original sand specimen and air to a three-phase medium comprising sand specimen, air, and water. The force between the particles decreased, and the compression deformation of the calcareous sand specimen decreased. When the particles were smaller, the contact area between the particles increased, the force between the particles weakened, the water in the internal pores could not be easily released, and the compression peak water content increased. Therefore, Specimen S had a higher water content than Specimen B.

**Figure 7:**
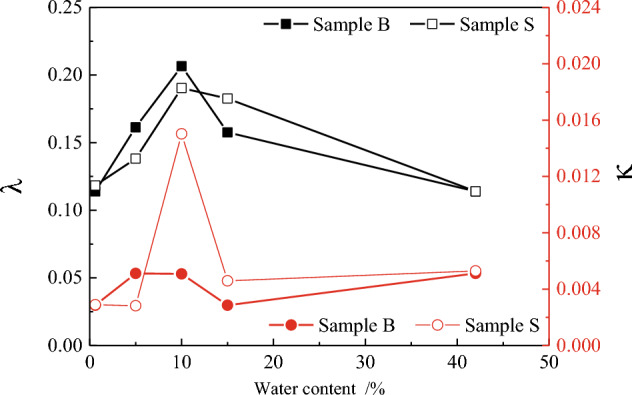
Water content versus the slope of the loading–unloading curve of calcareous sand.

As shown in Fig. 6, the calcareous sand specimen exhibited a very small rebound when unloading. This indicated that the compression deformation of the calcareous sand was mainly dominated by plastic deformation. The same experimental phenomenon has been reported in many studies^18,19^. To further study the compression–unloading characteristics, the compression–unloading curves under different water contents were obtained using the loading curve slope λ and unloading curve slope К of the normal consolidation line (NCL), similar to that in classical soil mechanics; Fig. 7 shows the results. With the increase in the water content, *λ* increased first and then decreased. When the water content was 10%, *λ* exhibited a high value, which was close to twice than that of dry sand. However, *К* had a considerably low variation. Therefore, the compression properties were closely related to the initial water content of the specimen. Since the compression deformation of the calcareous sand was dominated by plastic deformation, it had a considerably low effect on the resilience curve.

Under the same water content, the variations in *λ* and *К* of the specimens with different particle sizes were small or the same. This indicated that the posterior section of the compression curve and the unloading curve of the calcareous sand were approximately parallel to each other under the same water content. A similar phenomenon has been reported by Ghafghazi^32^ through tests. Meanwhile, this also indicated that the initial gradation had a considerably low effect on the compression and unloading characteristics of the specimen in the later stage of loading. This may be explained by the fact that with the increase in the pressure, the particle breakage of the sand specimens became increasingly serious, changing the particle gradation, and the initial gradation had an increasingly less effect on the calcareous sand.

**Figure 8:**
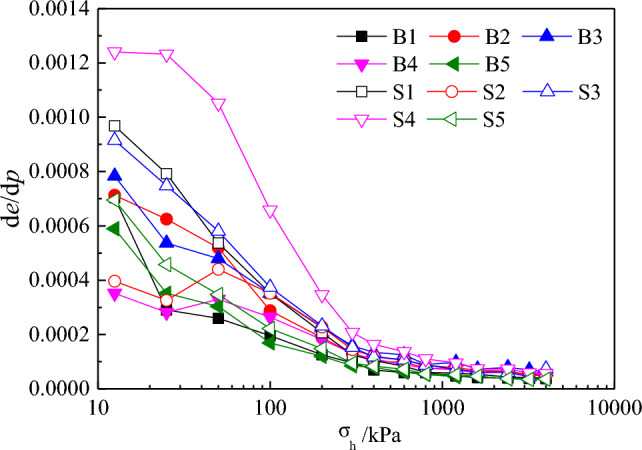
(− d*e*/d*p*)—*p* curves.

To thoroughly understand the effects of different water contents on the compression deformation under various levels of pressure *p*, the variations in the void ratio (− d*e*/d*p*) under various levels of pressure increments were plotted, as shown in Fig. 8. The effects of the water content on the compression coefficient of calcareous sand mainly occurred at low pressures and gradually reduced with increasing pressure. This may be explained by the fact that with the increase in the pressure, the void ratio gradually decreased, and after the density of the calcareous sand specimen increased to a certain level, particle movement and breakage became increasingly difficult, thus decelerating the compression deformation and reducing the differences in the variation in the void ratios.

Notably, the single-particle-size calcareous sand specimen selected in this study was only to increase the particle breakage of the specimen; it does not mean that this type of soil can be used in practical engineering. In practical engineering, the use of calcareous sand foundation is often a last resort. Because the special geographical environment is far away from the mainland and site condition constraints, in the construction of islands and reefs, a construction scheme involving local materials can only be adopted, using calcareous sand from a lagoon as the filling material and fencing the coral reef pads to serve as the foundations of buildings and structures. Therefore, sand with a single-particle-size gradation will not be specially selected for engineering construction. Meanwhile, this study mainly investigated the effects of water content on the particle breakage and compression characteristics of calcareous sand. The microscopic characteristics of the specimen, percolation characteristics, and pore pressure dissipation in the compression consolidation process are yet to be explored.

## Conclusions

In this study, confined compression tests were conducted on calcareous sand under different particle gradations and water contents. The compaction characteristics and the relationship between particle breakage and water content of the sand specimen were investigated. The main conclusions of this study are as follows:

1. With the increase in the water content, the final compression deformation of the calcareous sand gradually increased. However, when the water content reached a certain value, the final compression deformation decreased. The magnitude of this water content value was related to the initial gradation of the calcareous sand.
2. The compression deformation of calcareous sand was mainly dominated by plastic deformation. Under the same water content, the initial gradation had no effects on the compression and unloading characteristics of the specimen in the later stage of loading. The effects of water content on the compression coefficient of calcareous sand mainly occurred when the pressure was low, and with the increase in the pressure, this effect gradually weakened.
3. Under different water contents, the relative breakage index of the particles increased with increasing in confining pressure, and the empirical formula for calculating the breakage particle was obtained by fitting. The effects of the variation in the water content on the particle breakage of the calcareous sand were mainly reflected in the softening effect of water on the specimen particles, which reduced the Mohr strength of the particles, and this macroscopically reflected in the relative breakage index of the particles.

## Data Availability

The data used in this study can be provided by the corresponding author upon request.
